# Physiological and Transcriptome Analysis of Sugar Beet Reveals Different Mechanisms of Response to Neutral Salt and Alkaline Salt Stresses

**DOI:** 10.3389/fpls.2020.571864

**Published:** 2020-10-19

**Authors:** Gui Geng, Renren Li, Piergiorgio Stevanato, Chunhua Lv, Zhengyu Lu, Lihua Yu, Yuguang Wang

**Affiliations:** ^1^Heilongjiang Sugar Beet Center of Technology Innovation, College of Advanced Agriculture and Ecological Environment, Heilongjiang University, Harbin, China; ^2^Key Laboratory of Sugar Beet Genetic Breeding of Heilongjiang Province, College of Advanced Agriculture and Ecological Environment, Heilongjiang University, Harbin, China; ^3^College of Life Sciences, Heilongjiang University, Harbin, China; ^4^DAFNAE, Dipartimento di Agronomia, Animali, Alimenti, Risorse Naturali e Ambiente, Università degli Studi di Padova, Legnaro, Padua, Italy

**Keywords:** salt stress, sugar beet, physiological analysis, transcriptomic analysis, differentially expressed gene

## Abstract

The salinization and alkalization of soil are widespread environmental problems. Sugar beet (*B. vulgaris* L.) is a moderately salt tolerant glycophyte, but little is known about the different mechanisms of sugar beet response to salt and alkaline stresses. The aim of this study was to investigate the influence of neutral salt (NaCl:Na_2_SO_4_, 1:1) and alkaline salt (Na_2_CO_3_) treatment on physiological and transcriptome changes in sugar beet. We found that a low level of neutral salt (NaCl:Na_2_SO_4_; 1:1, Na^+^ 25 mM) or alkaline salt (Na_2_CO_3_, Na^+^ 25 mM) significantly enhanced total biomass, leaf area and photosynthesis indictors in sugar beet. Under a high concentration of alkaline salt (Na_2_CO_3_, Na^+^ 100 mM), the growth of plants was not significantly affected compared with the control. But a high level of neutral salt (NaCl: Na_2_SO_4_; 1:1, Na^+^ 100 mM) significantly inhibited plant growth and photosynthesis. Furthermore, sugar beet tends to synthesize higher levels of soluble sugar and reducing sugar to cope with high neutral salt stress, and more drastic changes in indole acetic acid (IAA) and abscisic acid (ABA) contents were detected. We used next-generation RNA-Seq technique to analyze transcriptional changes under neutral salt and alkaline salt treatment in sugar beet. Overall, 4,773 and 2,251 differentially expressed genes (DEGs) were identified in leaves and roots, respectively. Kyoto encyclopedia of genes and genomes (KEGG) analysis showed that genes involving cutin, suberine and wax biosynthesis, sesquiterpenoid and triterpenoid biosynthesis and flavonoid biosynthesis had simultaneously changed expression under low neutral salt or alkaline salt, so these genes may be related to stimulating sugar beet growth in both low salt treatments. Genes enriched in monoterpenoid biosynthesis, amino acids metabolism and starch and sucrose metabolism were specifically regulated to respond to the high alkaline salt. Meanwhile, compared with high alkaline salt, high neutral salt induced the expression change of genes involved in DNA replication, and decreased the expression of genes participating in cutin, suberine and wax biosynthesis, and linoleic acid metabolism. These results indicate the presence of different mechanisms responsible for sugar beet responses to neutral salt and alkaline salt stresses.

## Introduction

Soil salinization is one of the major environment problems that limit agricultural production worldwide and cause environmental hazards (Shabala et al., 2015). K^+^, Na^+^, Ca^2+^, Mg^2+^, Cl^–^, HCO_3_^–^, CO_3_^2–^, and SO_4_^2–^ are the main ions in natural soil. Soil salinization and alkalization frequently occur at the same time in nature (Zhang et al., 2018). Saline soils comprise high levels of neutral salts, which mainly include NaCl and Na_2_SO_4_, causing salt stress. However, alkalization of soils is related to alkaline salts (Na_2_CO_3_/NaHCO_3_) and associated with high pH (Yu et al., 2014). Stress resulting from saline soils induces osmotic stress and ion injury by disrupting ion homeostasis in plant cells (Yang and Guo, 2018a). Alkaline soils not only induce ion toxicity or injury but also CO_3_^2–^/HCO_3_^–^ stress and high pH stress. Thus, salt-alkaline stress causes several types of damage to plants as a result of salt ions and high pH (Yu et al., 2014). It is therefore urgent to understand the molecular and physiological mechanisms of plant response to saline-alkali stress. Elucidating the molecular mechanisms of plant adaptation would be useful for cultivating crops with strong salt and alkali tolerance.

To cope with saline-alkaline stress, plants have evolved a variety of strategies to adapt to the saline-alkali environments. In general, the adaptive strategies of plants to neutral salt stress mainly include accumulating of osmotic adjustment substances, enhancing the reactive oxygen species (ROS) scavenging system and maintaining ion homeostasis (Yang and Guo, 2018b; Lv et al., 2019). Many genes associated with these salt tolerant physiological and biochemical processes were identified through genomics or transcriptomic tools (Prerostova et al., 2017; Geng et al., 2019). However, insufficient information is available regarding alkaline tolerance-related genes and the mechanisms of plant alkaline salt tolerance. Knowledge about the difference between salt and alkaline stress is especially limited. A comparative analysis of the responses of plants to high salinity and alkalinity conditions should therefore be useful for identifying the genes or metabolic pathways specifically elicited by salt and alkaline stress.

Sugar beet (*Beta vulgaris* L.) is an important crop, which is primarily cultivated for extracting sugars from its tap root (Ji et al., 2019). It is not only used in the food industry, but also for production of bioethanol (Magaña et al., 2011). Sugar beet is considered a moderately salt tolerant plant as it can tolerate salinity of up to 500 mM sodium chloride (NaCl) for 7 days without losing viability (Yang et al., 2012). Recently, genome information on sugar beet has been released, making it good material for studying the mechanism of salt tolerance in plants (Dohm et al., 2013). However, the study on the mechanism of sugar beet salt tolerance mainly focuses on the physiological and molecular response changes in response to neutral salt (NaCl) (Hossain et al., 2017; Wang et al., 2019). Little is known about the influence of alkaline salt stresses. Few researches have been conducted to explore the different responses to neutral salt and alkaline salt stresses in sugar beet. In this study, we systematically analyzed the physiological and transcriptomic response of plants to neutral salt and alkaline salt. RNA-seq technology was applied to analyze the expression profiles of leaves and roots under neutral salt and alkaline salt stresses. The objectives of the study are identifying the different mechanisms of response to neutral and alkaline salt stress. This study provides new insights for understanding sugar beet tolerance to salt-alkaline stress.

## Materials and Methods

### Plant Materials and Salt Stress Treatment

The seeds of *Beta vulgaris* cv. H004 were used as the plant material in this experiment and were obtained from Advanta Company of Netherlands. The sugar beet seeds were sown in plastic pots contained 0.8 kg of soil (vermiculite: washed sands: black soil, 1:1:3), and the detail of the soil was listed in Supplementary Table 1. In this study, each pot was added 30 mL of one half Hoagland nutrient solutions and was sown with twenty seeds. The control group refers to the seeds sown in soil without salt. In the salt treatment group, seeds were planted in pots containing different kinds of salt. The salinity treatments used in this experiment were divided into neutral salt (NS) (NaCl and Na_2_SO_4_, at 1:1 Na^+^ molar ratio) and alkaline salt treatments (AS) (Na_2_CO_3_). The neutral salt or alkaline salt treatments contained two concentrations of each respective salt (total 25 mM Na^+^ and 100 mM Na^+^), resulting in a total of 4 kinds of treatment (NS25, NS100, AS25 and AS100) with varying salinity and pH (Table 1). After sowing, emergence was observed and recorded every day. Five days after sowing, four seedlings were selected and remained in each pot. Each pot was considered as a single replicate, and there were three replicates of each kind of salt treatment. All seedlings were cultivated in a growth chamber, with a 16 h/8 h photoperiod at 24°C/19°C (day/night) and 75% relative humidity at photoflux density of 450 μmol m^–2^ s^–1^. The plants in each pot were irrigated with 50 mL water every 4 days. After 25 days, all the seedlings were harvested. For transcriptomic or physiological analysis, roots or leaves of four seedlings from each treatment or control were pooled as a replicate, and each treatment group contained three independent biological replicates. Samples were frozen immediately in liquid nitrogen and stored at −80°C for physiological measurement and RNA extraction.

**TABLE 1:** pH and electrical conductivity (EC) of the neutral salt and alkaline salt treatment.

**Treatment**	**Name**	**Salinity (Na^+^ mM/kg soil)**	**EC (dS m^–1^)**	**pH**
No salt	Control (CK)	0	0.120 ± 0.011	6.19 ± 0.021
Neutral salt (NS)	NS25	25	2.65 ± 0.012	5.91 ± 0.013
	NS100	100	9.15 ± 0.024	5.45 ± 0.015
Alkaline salt (AS)	AS25	25	2.77 ± 0.022	8.82 ± 0.021
	AS100	100	8.89 ± 0.015	9.38 ± 0.019

*Data are the means of three biological replicates with standard deviation (SD).*

### Determination of Growth and Photosynthetic Indices

After harvesting leaf area was estimated by a LI-3000C portable area meter (LI-COR Biosciences, Lincoln, NE, United States). Total root length was determined by a root measuring system based on an optical scanner (WinRHIZO). Using a LI-6400 portable photosynthesis system (LI-COR Biosciences, Lincoln, NE, United States), net photosynthetic rate, stomatal conductance and intercellular carbon dioxide concentration were measured on harvest day (in the morning, 9:00 am). Chlorophyll content was measured using the method described by Kaur et al. (2016). 100 mg of fresh leaves were homogenized in liquid nitrogen. Then 1.5 mL of 80% acetone was added and the mixture was incubated in the dark for 1.5 h. The homogenate was centrifuged at 16,000 r min^–1^ for 4 min. The supernatant was collected and absorbance was measured spectrophotometrically at 645 and 663 nm against 80% acetone as blank.

### Measurement of Indole Acetic Acid (IAA), Abscisic Acid (ABA) and Gibberellic Acid (GA)

The IAA, ABA, and GA contents were measured by enzyme-linked immunosorbent (ELISA) kits provided by China Agricultural University (Wang et al., 2017). Fresh leaves (1 g) were homogenized in liquid nitrogen and hormones were extracted by 2 mL extraction buffer (80% methanol with 1 mM butylated hydroxytoluence). The mixture was stored at 4°C for 4 h and centrifuged at 3,500 r min^–1^ for 8 min at 4°C. The resulting supernatant was treated with C-18 solid phase extraction column, and the analyses were eluted from the column by 2.5 mL 100% (v/v) methanol from the column. The extract was vacuum evaporated to remove methanol, and the evaporated residue was dissolved with 2ml PBS buffer containing Tween 20 (0.1% [v/v]) and 1 g L^–1^ gelatin. ELISA measurement was performed on a 96-well microtitration plate provided by the manufacturer. Each well had 50 μL of extracts or standards added, and 50 μL of 5 μg mL^–1^ antibodies against GA, IAA, and ABA, respectively. The plate was incubated for 1 h at 37°C, and then washed four times with washing buffer (PBS buffer containing 0.1% Tween 20). 100 μL of 1.25 μg mL^–1^ IgG-horseradish peroxidase substrate was added to each well and the reaction lasted 30 min at 30°C. The microtitration plate was washed four times as above, and then 100 μL color-appearing solution containing 1 mg mL^–1^ *O*-phenylenediamine and 0.008% (v/v) H_2_O_2_ was added to each well. The reaction was stopped by adding 50 μL of 2M H_2_SO_4_. The OD values at 490 nm of each sample were determined by ELISA Reader.

### Determination of Sucrose and Reducing Sugars

Leaf tissue (0.1 g, dry weight) was used to extract soluble sugar in 7 mL of 80% ethanol and repeated three times at 80°C for 1.5 h. Ethanol was evaporated in the boiled water bath. Then 5 mL of 0.15% anthrone solution was added. The reaction mixture was incubated at 95°C for 20 min and left at room temperature for 20 min. The absorbance was measured at 620 nm. The content of soluble sugar was estimated using a standard curve. To assess the reducing sugars, the DNSA reagent (1 mL 3, 5-dinitrosalicylic acid) was mixed with ethanol extract (1 mL). The reaction mixture was heated for 12 min, and then 2 mL of distilled water was added (Jawad Hassan et al., 2020).

### Proline and Free Amino Acid Content Measurement

Proline content was determined using the method given by Bates et al. (1973). Leaf tissue (0.1 g, dry weight) was homogenized in 3% aqueous sulfosalicyclic acid and centrifuged at 12000 r min^–1^ for 15 min. 2 mL of supernatant was then mixed with 2 mL acid ninhydrin and 2 mL glacial acetic acid, and the mixture was incubated for 30 min at 95°C. 4 mL of toluene was next added to the reaction mixture with full shaking, and the proline content was determined by measuring absorbance of the toluene at 520 nm. The free amino acid contents were estimated by detecting the absorbance at 570 nm. The amount of free amino acid was determined using the amino acid standard curve (Jawad Hassan et al., 2020).

### cDNA Libraries Preparation and RNA-Seq

Total RNA was extracted from root and leaves using TRIzol Reagent (Invitrogen, Carlsbad, CA, United States) according to the manufacturer’s instructions, and RNA was digested with DNaseI (TaKaRa, Japan) at 37°C for 30 min. The quantity and quality of total RNA were determined by NanoDrop ND2000 spectrophotometer (NanoDrop Technologies, Wilmington, DE, United States) and 2100 Bioanalyser (Agilent), and the poly (A) mRNA was enriched through the oligo (dT) magnetic bead method. The cDNA library for transcriptome sequencing was constructed following TruSeq^TM^ RNA sample preparation kit from Illumina (San Diego, CA, United States). A total of thirty RNA samples were used to construct the transcriptome sequence library and each library was prepared using 5 μg of total RNA. After quantifying by TBS380, the thirty paired-end libraries were sequenced using Illumina HiSeq PE 2 bp × 151 bp high-throughput sequencing platform.

### Identification of Differentially Expressed Genes (DEGs) and Functional Analysis

Raw data generated by the sequencing platform was subjected to quality control (QC) to remove low-quality sequences, which include reads with adapter contamination, reads <70 bp after removing adapter sequences, reads containing more than 8% unknown bases, and reads in which >50% of the bases have a quality score <5. The clean reads were then mapped to the reference sequence (RefBeet-1.2.2) using Hisat2^1^. The expression of each gene was calculated by FRKM (Fragments Per Kilobase of exon model per Million mapped reads). The Cuffdiff^2^ software was used to identify genes that were differentially expressed between two samples. The false discovery rate (FDR) was used to determine the threshold of the p-value. Genes with FDR ≤ 0.05, | log_2_(fold change)| ≥ 1 and FRKM ≥ 1 were considered as significant differentially expressed genes (DEGs).

To investigate the function and biological pathways involved in the differentially expressed genes, Gene Ontology (GO) functional and Kyoto encyclopedia of genes and genomes (KEGG) pathway enrichment analyses were conducted by Goatools^3^ and KOBAS^4^ (Mao et al., 2005). DEGs were significantly enriched in metabolic pathways when their Bonferroni-corrected p-value was less than 0.05.

### Validation of DEGs Expression With Quantitative Real-Time PCR (qRT-PCR)

In order to evaluate the reliability of RNA-Seq experiments, six genes were selected (three in roots and leaves) to validate the transcriptome analysis using qRT-PCR. Total RNA from the roots and leaves of sugar beet was extracted with TRIzol reagent (Invitrogen, Carlsbad, CA, United States) and cDNA was synthesized using a reverse transcription kit from TOYOBO (Japan) according to the manufacturer’s instructions. The qRT-PCR analysis was performed using Bio-Rad Quantitative PCR System (Bio-Rad, Hercules, CA, United States) and the SYBR Green Real-time PCR Master Mix Kit (TOYOBO, Osaka, Japan). PCR amplification was performed under the following conditions: 20 s at 95°C, followed by 40 cycles of 95°C for 15 s, 55°C for 30 s and then 72°C for 20 s. Three biological replicates were carried out in each reaction, and normalization was done using *18S rRNA* gene as an internal control gene (Wang et al., 2019). All primer sequences were designed using PRIMER5 software and are listed in Supplementary Table 2.

### Statistical Analysis

The data obtained were expressed as means and standard errors, and all of the experiments were repeated three times. Analysis of variance (ANOVA) between the physiological parameters of plants in the control and salt treatments were performed using SPSS 13.0 (SPSS Inc., Chicago, IL, United States). The qRT-PCR data of the 6 genes were subjected to ANOVA analyses.

## Results

### Phenotypic and Physiological Response to Neutral Salt and Alkaline Salt Treatments

**FIGURE 1:**
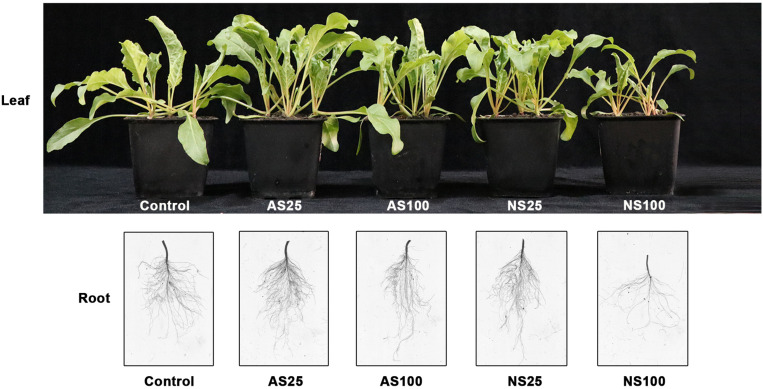
Morphological change in sugar beet under neutral salt (NS) and alkaline salt (AS) treatment.

**FIGURE 2:**
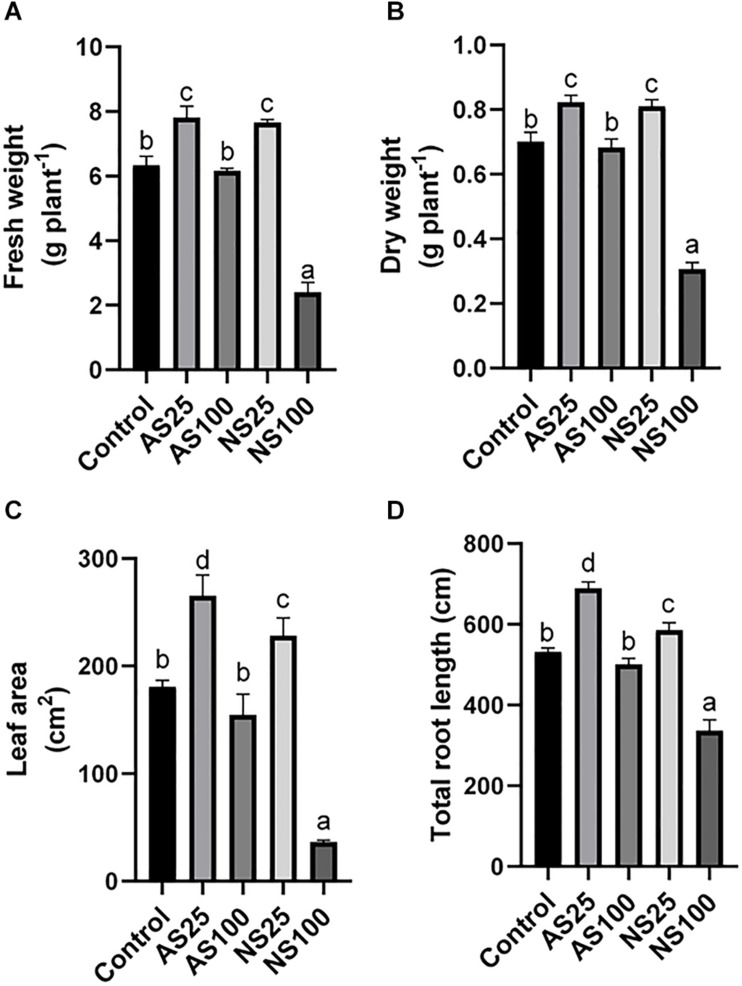
Effects of neutral salt (NS) and alkaline salt (AS) on fresh weight **(A)**, dry weight **(B)**, leaf area **(C)**, and total root length **(D)** of sugar beet. Values represent the means of three biological replicates. Different letters indicate significantly different at *p* < 0.05.

Sugar beet exposed to four kinds of salt treatment for 25 days showed different phenotypes and growth status (Figure 1). A low level of neutral salt and alkaline salt (NS25 and AS25) promoted the growth of seedlings. Under a high concentration of alkaline salt (As100), the growth of plants was not significantly affected. An obvious growth inhibition occurred under a high level of neutral salt (NS100), although the electrical conductivity of the two kinds of salt treatment is similar (Table 1). Consistently, the dry weight, fresh weight, leaf area and the total root length of seedlings increased significantly in NS25 and AS25 as compared to the control group (Figures 2A–D). In AS100, the dry weight, fresh weight, leaf area and total root length of seedlings had no significant change compared with the control group. However, these indexes decreased obviously in NS100 compared with the control group (Figure 2).

**FIGURE 3:**
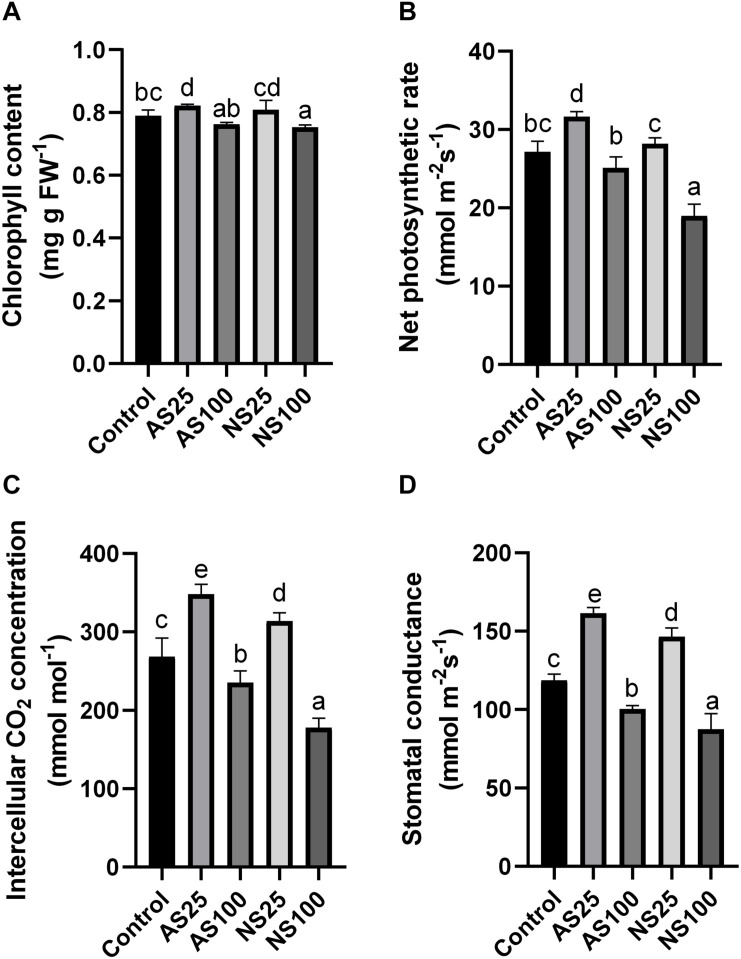
Effects of neutral salt (NS) and alkaline salt (AS) on chlorophyll content **(A)**, net photosynthetic rate **(B)**, intercellular CO_2_ concentration **(C)**, and stomatal conductance **(D)** in sugar beet. Values represent the means of three biological replicates. Different letters indicate significantly different at *p* < 0.05.

Several photosynthesis-related indicators were also detected to evaluate the response of sugar beet to neutral salt and alkaline salt. Chlorophyll content only showed a significant increase in As25 (Figure 3A). Net photosynthetic rate, intercellular CO_2_ concentration and stomatal conductance increased significantly in both NS25 and AS25 (Figures 3B–D). The photosynthetic rate in AS100 had no significant change as compared with the control, but photosynthetic rate reduced by 31.3% in NS100. High level of alkaline and neutral salt significantly reduced intercellular CO_2_ concentration and stomatal conductance. However, the effect of neutral salt stress was more significant compared with alkaline salt treatment. Our results showed that low concentration of neutral salt and alkaline salt can promote the photosynthesis of sugar beet seedlings. A high level of neutral salt stress exerted strongly negative effects on photosynthesis compared with high alkaline salt.

Plants can synthesize and accumulate osmotic adjustment molecules to maintain cell turgor and osmotic equilibrium in different compartments, especially when they are exposed to salt stress (Wang et al., 2013; Sui et al., 2015). In this study, the levels of proline and free amino acids in sugar beet leaves showed significant decreasing in AS25 and no change in AS100, NS25, and NS100 (Table 2). The levels of soluble sugar and reducing sugar in the leaves of sugar beet increased significantly in NS100 compared with the control, but the contents of soluble sugar and reducing sugar in AS100 were not changed (Table 2). Therefore, sugar beet tends to synthesize higher levels of soluble organic solutes to cope with neutral salt stress.

**TABLE 2:** Amounts of soluble sugar, reducing sugar, free amino acids, proline occurring in leaves of sugar beet treated with different level neutral salt (NS) or alkaline salt (AS).

	**Soluble sugar (mg g DW^–1^)**	**Reducing sugar (mg g DW^–1^)**	**Proline (μmol g DW^–1^)**	**Free amino acids (μmol g DW^–1^)**
Control	3.233 ± 0.096^*c*^	26.577 ± 0.105^*b*^	1.750 ± 0.163^*b*^	54.717 ± 0.834^*b*^
AS25	2.391 ± 0.052^*a*^	20.000 ± 1.001^*a*^	1.359 ± 0.149^*a*^	46.363 ± 0.287^*a*^
AS100	3.485 ± 0.249^*c*^	25.839 ± 1.967^*b*^	1.772 ± 0.072^*b*^	55.550 ± 3.249^*b*^
NS25	2.877 ± 0.106^*b*^	24.113 ± 0.440^*b*^	1.630 ± 0.108^*b*^	55.963 ± 1.339^*b*^
NS100	3.797 ± 0.225^*d*^	29.130 ± 2.760^*c*^	1.735 ± 0.066^*b*^	52.954 ± 1.681^*b*^

*Means ± SD (three biological replicates) with different letters are significantly different at *p* < 0.05.*

As the response of plants to salt stress is closely influenced by plant hormone signaling, the influence of neutral and salt stress on the levels of GA, ABA, and IAA were examined in sugar beet (Table 3). Both low neutral salt and alkaline salt led to increasing the GA contents. Moreover, the production of IAA was significantly lower in NS100 than that under control conditions, and there was no significant difference in leaf content between AS100 and the control group. The decrease of IAA content caused by high concentration of neutral salt may lead to the inhibition of sugar beet growth. Moreover, the level of ABA increased remarkably in NS100 and AS100 compared with the control group, and the increase of ABA in NS100 was significantly higher than that in AS100 (46.3 *vs.* 26.9%). Thus, neutral salt causes more drastic changes in ABA level.

**TABLE 3:** The levels of indole acetic acid (IAA), abscisic acid (ABA), and gibberellic acid (GA) in leaves of sugar beet treated with different level neutral salt (NS) or alkaline salt (AS).

	**IAA (ng g FW^–1^)**	**ABA (ng g FW^–1^)**	**GA_1__+__3_ (ng g FW^–1^)**
Control	85.120 ± 0.917^*b*^	41.951 ± 3.750^*a*^	13.506 ± 0.137^*b*^
AS25	96.852 ± 2.925^*b*^	37.732 ± 0.823^*a*^	14.604 ± 0.384^*c*^
AS100	76.293 ± 5.145^*ab*^	53.238 ± 1.269^*b*^	12.985 ± 0.179^*a*^
NS25	87.718 ± 1.582^*ab*^	38.620 ± 1.658^*a*^	14.396 ± 0.417^*c*^
NS100	69.782 ± 3.248^*a*^	61.407 ± 3.184^*c*^	12.406 ± 1.022^*ab*^

*Means ± SD (three biological replicates) with different letters are significantly different at *p* < 0.05.*

### Transcriptome Sequencing and Alignment

To obtain a comprehensive overview of the sugar beet transcriptome pattern in response to neutral and alkaline salt, RNA-Seq analyses were conducted on the seedlings under neutral and alkaline salt treatment. Between 39 and 64 million 150 bp paired-end raw reads approximately were obtained for each library, and the Q30 of the base ratio was higher than 92.25% (Supplementary Table 3). After removing adapter and low-quality reads, clean reads were mapped to the *Beta Vulgaris* referenced genome, and between 87.96 and 92.63% of the clean reads were mapped to the reference database in each of the thirty libraries (Supplementary Table 3). These results indicated that the quality of sequencing data is robust for subsequent analysis.

### Analysis of Differentially Expressed Genes Under Neutral or Alkaline Salt Treatments

**FIGURE 4:**
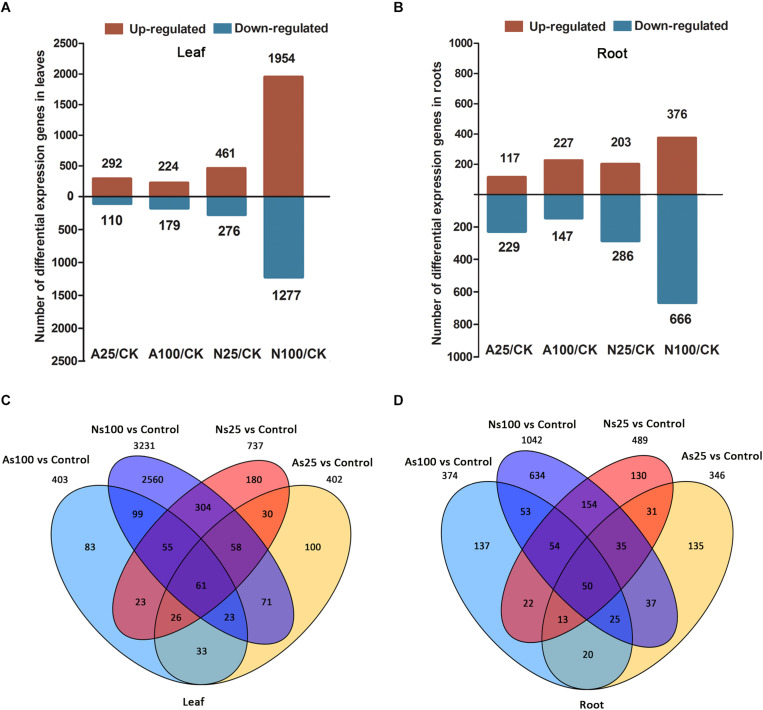
Differentially expressed genes (DEGs) in the leaves and roots of sugar beet in response to neutral salt (NS) and alkaline salt (AS) treatment. Numbers of DEGs in leaf **(A)** and root **(B)** at different salt treatments. Venn diagrams of DEGs among different salt treatments in leaf **(C)** and root **(D)**.

To identify differentially expressed genes (DEGs) during each salt treatment, we compared the expression level of each gene between salt treatment and control samples. The expression of each gene was calculated by fragments per kilobase of exon model per million fragments mapped (FPKM) based on the gene length and its mapped fragments count. All annotated genes that were used to further identify DEGs in sugar beet response to neutral and alkaline salt treatment are listed in Supplementary Tables 4, 5. The differentially expressed genes (DEGs) under salt treatment, were selected according to a threshold FDR < 0.05, fold change ≥2 (log_2_ fold change (FC) ≥ 1 or log_2_ FC ≤ -1 and FPKM ≥ 1) between the three salt-treated and control samples. This analysis revealed variations between different salt treatments (Figure 4). Compared with the control, a total of 402, 403, 737, and 3231 genes were identified to be differentially regulated in the leaves of AS25, AS100, NS25, NS100, respectively (Figure 4A and Supplementary Table 6). Moreover, we found that there were 346, 374, 489, and 1042 DEGs for roots in AS25, AS100, NS25, NS100 (Figure 4B and Supplementary Table 7). DEGs at different salt treatments were therefore dramatically different. For example, higher numbers of DEGs were observed in the comparisons of NS100 with control in leaves (1954 up and 1277 down), but the lowest number of DEGs was found in the comparison of AS100 with the control in leaves (224 up and 179 down) (Figure 4A). Thus, in sugar beet, neutral salt stress elicits greater transcriptomic changes than alkaline stress and it results in a higher number of DEGs compared to the latter. Furthermore, overlapping studies found that there were 100, 83, 180, and 2560 unique genes in the leaves of AS25, AS100, NS25, NS100, and a total of 135, 137, 130, 634 DEGs were unique for each salt treatment in roots, respectively (Figures 4C,D). These DEGs may have contributed to the phenotypic differences in different salt treatments.

### GO Enrichment Analysis for the DEGs in Response to Neutral Salt and Alkaline Salt

To investigate possible biological functions of DEGs that determine the response of sugar beet under different salt treatments, GO enrichment analysis were conducted and DEGs were assigned to GO terms in three ontologies involved in cellular components, biological processes and molecular function (Supplementary Tables 8, 9). Based on the GO enrichment results, catalytic activity (GO:0003824), transmembrane transporter activity (GO:0015144), glucosyltransferase activity (GO:0046527) and hydrolase activity (hydrolyzing O-glycosyl compounds) (GO:0004553) were the most dominant terms in the molecular function category for the leaves of AS25, AS100, NS25, NS100, respectively (Supplementary Table 8). Moreover, inorganic anion transmembrane transporter activity (GO:0015103) were identified as the most dominant GO terms in molecular function category for roots in AS25, AS100, and NS100, but glucosyltransferase activity (GO:0046527) were found as the most dominant terms in the roots of NS100 (Supplementary Table 9). In cellular component category, extracellular region (GO:0005576), cell wall (GO:0005618), and external encapsulating structure (GO:0030312) were all significantly enriched in the roots and leaves of AS25, AS100, NS25, NS100 (Supplementary Tables 8, 9).

### KEGG Enrichment Analysis for the DEGs in Response to Neutral Salt and Alkaline Salt

**FIGURE 5:**
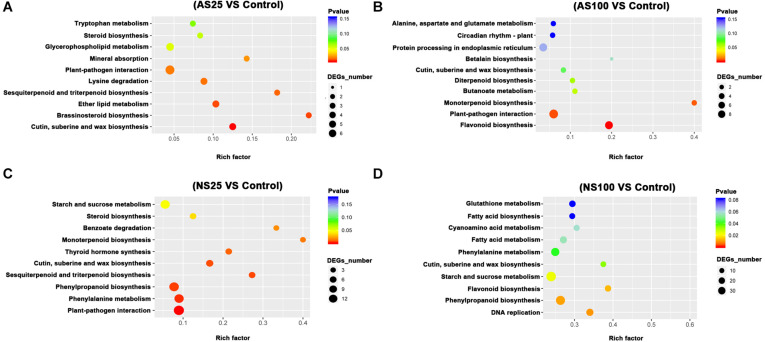
The KEGG (Kyoto Encyclopedia of Genes and Genomes, http://www.genome.jp/kegg) pathway enrichment analysis of DEGs (differentially expressed genes) in the leaves of sugar beet in response to neutral salt (NS) and alkaline salt (AS) treatment. KEGG enrichment pathways in the leaves of sugar beet at AS25 (alkaline salt, 25 mM Na^+^) **(A)**, AS100 (alkaline salt, 100 mM Na^+^) **(B)**, NS25 (neutral salt, 25 mM Na^+^) **(C)**, and NS100 (neutral salt, 100 mM Na^+^) **(D)**.

**FIGURE 6:**
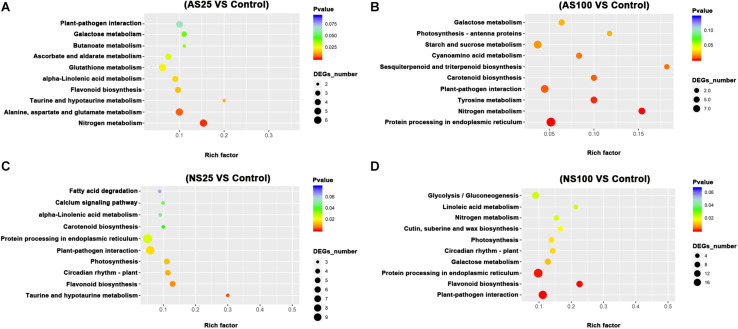
The KEGG (Kyoto Encyclopedia of Genes and Genomes, http://www.genome.jp/kegg) pathway enrichment analysis of DEGs (differentially expressed genes) in the roots of sugar beet in response to neutral salt (NS) and alkaline salt (AS) treatment. KEGG enrichment pathways in the roots of sugar beet at AS25 (alkaline salt, 25 mM Na^+^) **(A)**, AS100 (alkaline salt, 100 mM Na^+^) **(B)**, NS25 (neutral salt, 25 mM Na^+^) **(C)**, and NS100 (neutral salt, 100 mM Na^+^) **(D)**.

In order to elucidate the major biological pathways that are affected by different salt treatments, we performed a gene enrichment analysis of the DEGs based on the KEGG to identify significantly enriched biological pathways. KEGG enrichment analysis revealed that different salt treatments led to the enrichment of DEG_*S*_ in different KEGG pathways (Figures 5, 6). For example, pathways involved in flavonoid biosynthesis, plant-pathogen interaction and monoterpenoid biosynthesis were significantly enriched among the DEGs of leaves in AS100 (Figure 5). However, DNA replication, phenylpropanoid biosynthesis, flavonoid biosynthesis, starch and sucrose metabolism, cutin, suberine and wax biosynthesis, and phenylalanine metabolism were significant enrichment pathways among DEG_*S*_ in leaves at NS100 treatment. In addition, the KEGG pathway enrichment analysis of DEG_*S*_ in roots under different salt treatments was also performed (Figure 6). Compared with the pathway enriched in leaves, several KEGG pathways were specially enriched in roots, such as protein processing in endoplasmic reticulum, nitrogen metabolism and tyrosine metabolism (Figure 6). In summary, the DEG_*S*_ in sugar beet were enriched to different metabolic pathways under different salt treatments that may explain the phenotypic differences in sugar beet under neutral and alkaline salt treatment.

### Pathways Related to Sugar Beet Response to Different Level of Neutral Salt and Alkaline Salt

**FIGURE 7:**
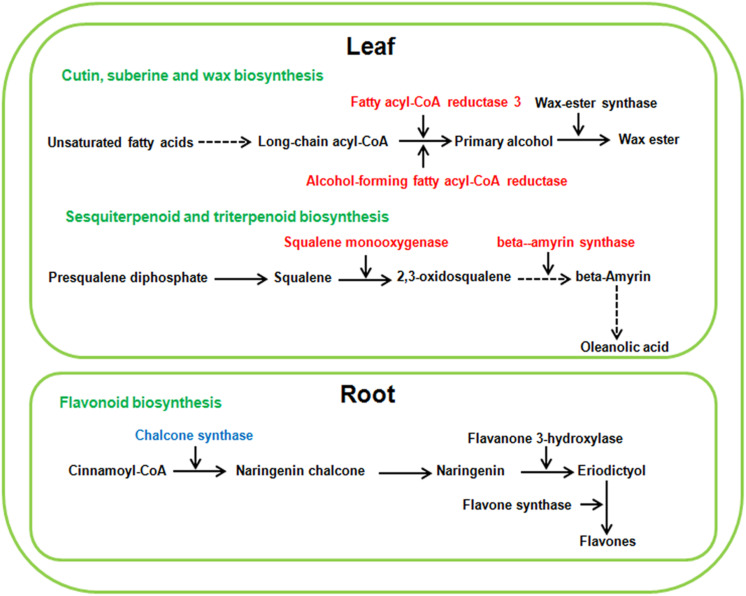
Schematic presentation of the potential mechanism of low neutral salt or alkaline salt enhanced sugar beet growth. Red and blue highlighted genes indicate simultaneous increase or decrease in both low neutral salt and alkaline salt.

As a low level of neutral salt and alkaline salt (NS25 and AS25) can enhance the growth of sugar beet seedlings, the enrichment pathways of DEG_*S*_ at NS25 and AS25 treatments were compared in our study. Cutin, suberine and wax biosynthesis, sesquiterpenoid and triterpenoid biosynthesis were simultaneously enriched in DEG_*S*_ of leaves at AS25 and NS25 treatment (Figure 5). Several DEG_*S*_ involved in cutin, suberine and wax biosynthesis, and sesquiterpenoid and triterpenoid biosynthesis were up-regulated in the leaves of NS25 and AS25 (Figure 7 and Supplementary Table 10). Furthermore, we found that the pathway of flavonoid biosynthesis was enriched among DEG_*S*_ of roots in NS25 and AS25 (Figure 7 and Supplementary Table 10). For example, *chalcone synthase* (LOC104902217) was down-regulated in the roots of NS25 and AS25 (Figure 7). Therefore, these pathways may participate in the stimulation of sugar beet growth by a low level of neutral salt and alkaline salt.

**FIGURE 8:**
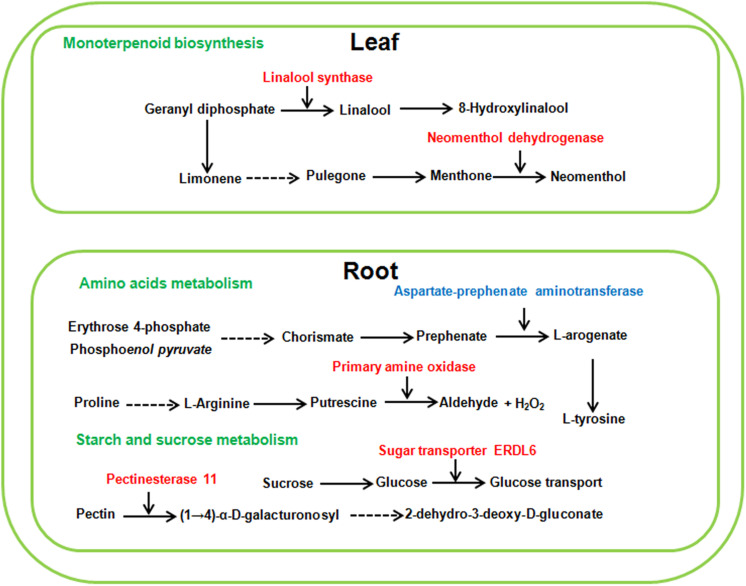
Schematic presentation of the potential mechanism of sugar beet alkaline salt tolerance. Red and blue highlighted genes indicate specifically increased or decreased in high alkaline salt compared with high neutral salt.

Because sugar beet showed different phenotype and physiological changes under high concentration of neutral salt and alkaline salt, we hypothesized that some metabolic pathways with specific enrichment of DEG_*S*_ in AS100 may be involved in the alkaline salt tolerance phenotype of sugar beet. In this study, the pathway of monoterpenoid biosynthesis was specifically enriched in leaves at AS100 treatment, meanwhile, amino acids metabolism and starch and sucrose metabolism were specifically found in the roots at AS100 treatment compared with NS100 (Figure 8 and Supplementary Table 11). Several genes involved in these metabolic pathways were found to be differentially expressed in sugar beet treated with a high concentration of alkaline salt (Figure 8 and Supplementary Table 11).

**FIGURE 9:**
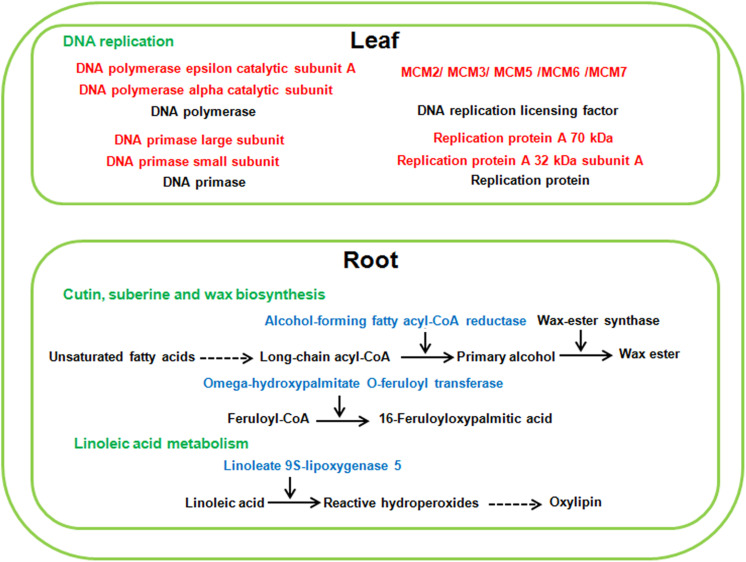
The specific pathways of sugar beet response to high neutral salt stress. Red and blue highlighted genes indicate specifically increased or decreased in high neutral salt compared with high alkaline salt.

Compared with alkaline salt treatment, a high level of neutral salt (NS100) obviously inhibited the growth status of sugar beet. Therefore, several genes may be only differentially expressed in response to a high level of neutral salt environment. It is noteworthy that pathways involved in DNA replication were only enriched significantly in leaves under NS100 condition compared with AS100. Moreover, pathways related to cutin, suberine and wax biosynthesis, and linoleic acid metabolism were only identified in roots at NS100 (Figure 9 and Supplementary Table 12). We speculated that a high level of neutral salt affected the expression of genes related to these metabolic pathways and induced specific response to high neutral salt stress response, leading to the phenotype changes.

### Validation of RNA-Seq Analysis Using Quantitative RT-PCR

**FIGURE 10:**
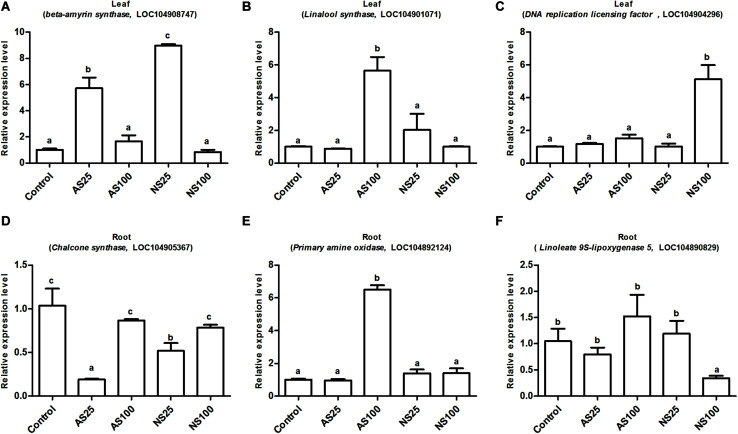
Expression level of the selected six genes by qRT-PCR (quantitative real-time PCR) validation. **(A,D)** Indicate differentially expressed genes (DEGs) involved in low neutral salt or alkaline salt enhanced sugar beet growth. **(B,E)** Indicate DEGs involved in sugar beet alkaline salt tolerance. **(C,F)** Indicate DEGs involved in specific pathways of sugar beet response to high neutral salt stress. Values represent the means of three replicates. Different letters indicate significantly different at *p* < 0.05. Three biological replicates were performed.

To validate the reliability of the expression profiles obtained by RNA-Seq, six candidate DEG_*S*_ were selected for qRT-PCR assays. The six genes were involved in the metabolic pathways mentioned obviously (Figures 7–9). Similar expression patterns were found for all selected genes, although the fold-changes detected by RNA-Seq and qRT-PCR did not match perfectly (Figure 10 and Supplementary Tables 6, 7). For example, qRT-PCR analyzes found the DNA replication licensing factor showed significant increase in transcript level, which was consistent with transcriptome data (Figure 10 and Supplementary Table 6). These results indicate that the RNA-Seq data were largely reliable and truly reflected gene expression in response to salt stress.

## Discussion

### Phenotypic and Physiological Response to Neutral Salt and Alkaline Salt Treatments

Sugar beet is a glycophyte with strong salt tolerance. Several studies on exploring and comparing the morpho-anatomical responses of plants to alkaline and salt stress have been conducted, but few have investigated the sugar beet response to salt and alkaline stress. Sugar beet inherited the salt tolerance trait from its ancestor sea beet (*B. vulgaris* ssp. *Maritima*), which grows naturally along the Atlantic coasts of Western Europe (Lv et al., 2019). We consistently found that low concentrations of neutral and alkaline salts stimulated growth. As the net photosynthetic rate, intercellular CO_2_ concentration and stomatal conductance increased significantly in low levels of neutral and alkaline salts, enhancing photosynthesis is involved in stimulating the growth. In comparison with the situation under high levels of neutral and alkaline salts, seedlings suffered more changes under neutral salt stress. For example, a high concentration of neutral salt caused severe growth inhibition (Figure 1) and a sharp decline in photosynthetic indices, but these parameters were not significant affected by a high concentration of alkaline salt (Figure 3). Moreover, the high level of neutral salt can also cause drastic change in hormone content as well as some osmotic adjustment substance content (Tables 2, 3). This study indicated that sugar beet showed stronger alkaline salt resistance compared with neutral salt. Our results are in contrast with previous studies on other plants, which found that injurious effects of alkali stress on plants are more severe than those of salt stress (Guo et al., 2017). The injurious effect of neutral salt is commonly caused by osmotic stress and ion toxicity. By contrast, alkaline salt stress involves high-pH stress, in addition to these two stress factors. Furthermore, high pH also caused decline of protons and the destruction of transmembrane electrochemical potential gradients in cells (Paz et al., 2014). Therefore, this result implied that sugar beet had a distinct adaptive mechanism to cope with alkaline salt stress.

### DEGs Related to Sugar Beet Response to Different Level of Neutral Salt and Alkaline Salt

RNA-seq analysis of roots and leaves under neutral and alkaline salts stresses was performed to investigate different molecular mechanisms under salt and alkaline stress. From our analysis, we found many genes related to different phenotypes under different neutral salt and alkaline salt treatments.

#### DEG_*S*_ Involved in the Stimulation of Sugar Beet Growth by Low Level Neutral and Alkaline Salts

Several genes involved in cutin, suberine and wax biosynthesis, sesquiterpenoid and triterpenoid biosynthesis were simultaneously up-regulated in leaves treated with low level neutral and alkaline salt. The cuticle is a continuous hydrophobic layer that coats most aerial surfaces of terrestrial plants (Rowland et al., 2006). The main function of the cuticle is to restrict non-stomatal water loss and repel rainwater. It was also related to protecting the plant from desiccation, UV light and pathogens (Lee and Suh, 2015). Structurally, the cuticle consists primarily of cutin and waxes. Cuticular waxes are complex mixtures of very long chain fatty acids, primary and secondary alcohols, esters and so on. Fatty acyl-CoA reductase (FAR) is the key enzyme in the process of wax synthesis, which reduces fatty acyl-CoAs to primary alcohol. It was found that drought stress induced the expression of *TaFAR1* in wheat (Wang et al., 2015). Moreover, the up-regulation of *TaFAR1* leads to an accumulation of primary alcohol in cuticular wax. In our study, low levels of neutral and alkaline salt all induced the expression of two *FAR* genes (LOC104901470; LOC104900340) in leaves of sugar beet, respectively. Thus we speculated that the accumulation of primary alcohol in cuticular wax was involved in growth stimulation under a low level of salt treatment.

Triterpenoids, containing carbon skeleton based on six isoprene units, constitute one of the largest groups of secondary metabolites in plants (Shang and Huang, 2019). Triterpenoids are implicated in various plant processes, including respiration, photosynthesis and the response to both environmental stimuli and biotic stress challenges (Laranjeira et al., 2015). 2,3-oxidosqualene is an important compound in the early steps of triterpenoid biosynthesis in plants. Squalene monooxygenase has been identified as a fundamental enzyme converting squalene into 2,3-oxidosqualene. Moreover, for oleanolic acid biosynthesis, 2,3-oxidosqualene is cyclized to the pentacyclic oleanane-type triterpenoid backbone bamyrin by the b-amyrin synthase (BAS) (Abe, 2007). In this study, *squalene monooxygenase* (LOC104908747) and *b-amyrin synthase* (LOC104901642) were induced to express in the leaves of sugar beet under low concentration of neutral salt and alkaline salt. Thus, the biosynthesis of triterpenoids and oleanolic acid may be related to promoting growth.

Flavonoids are the major component of plant polyphenolic secondary metabolites, and they perform a wide range of functions, such as antioxidant activity, UV-light protection and defense against phytopathogens (Petrussa et al., 2013). However, it is found that several kinds of flavonoid could negatively regulate plant tolerance to salinity (Pi et al., 2019). In our study, the expression of *chalcone synthase* (LOC104902217) and *flavonol synthase* (LOC104883093) were down-regulated in root under a low level of alkaline salt treatment, and *chalcone synthase* (LOC104902217; LOC104887632; LOC104887630) and *cytochrome P450* (LOC104886776) decreased the expression in root under a low level of neutral salt treatment (Supplementary Table 8). These changes may lead to a lower accumulation of several kinds of flavonoid at low salt level, which negatively regulated plant salt stress tolerance.

#### DEG_*S*_ Involved in the Sugar Beet Tolerance to High Level Alkaline Salt

Plant monoterpenoids belong to a large family of plant secondary metabolites and are crucial for many biological activities of plants, such as defense against herbivores, pollination and stress signal transduction (Mendoza-Poudereux et al., 2014). Geranyl diphosphate (GPP) is the universal precursor of monoterpenoids. The GPP is further transformed into diverse monoterpenoids (Degenhardt et al., 2009). Linalool synthase catalyzes the conversion of GPP to linalool, which is an important monoterpene substance. It was found that transgenic sweet orange (*Citrus sinensis* L. Osbeck) plants over-expressing a *linalool synthase* gene led to the accumulation of linalool. Moreover, transgenic plants exhibited strong resistance to citrus canker and up-regulation of defense-related genes (Shimada et al., 2017). In this study, we found a special significant up-regulation of *linalool synthase* (LOC104901071) under high level of alkaline salt stress in sugar beet leaf. The induced expression of *linalool synthase* may cause the high level of linalool and lead sugar beet to adapt to alkaline salt stress. In addition, neomenthol dehydrogenase (LOC104888569), an important enzyme in the metabolism of monoterpenes, is also specifically induced to express under alkaline salt stress in leaf. Neomenthol dehydrogenase participates in the conversion L-menthone to neomenthol (Davis et al., 2005). It is not clear whether neomenthol is involved in plant response to salt stress. Overall, the induced expression genes participating in monoterpenoids biosynthesis may make a concerted effort for adaptation of plants to high levels of alkaline salt stress.

Starch and sucrose metabolism products have many roles in higher plants; in particular, they are important for seed germination, shoot growth and transient carbon reserves. It was previously reported that starch and sucrose metabolism was involved in the process of plant response to salt stress (Sui et al., 2015). For example, starch and sucrose might be pivotal for salt tolerance of *Thellungiella halophila*, which is a model halophyte used to study plant salt tolerance (Wang et al., 2013). After treatment with high level of alkaline salt, the expression of DEGs related to starch and sucrose metabolism were enhanced in the roots of sugar beet. In this study, the expression of pectinesterase 11 (LOC104890721) was only found to increase in the roots of AS100 compared with that of NS100. Pectinesterase acts in the modification of cell walls via demethylesterification of cell wall pectin (Yan et al., 2018). Recently a link has been established between *Pectinesterase 31* (*PME31*) and salt stress tolerance. Salt stress significantly increased *PME31* expression, and knock-down mutants in *PME31* adapted hypersensitive phenotypes to salt stress (Yan et al., 2018). Furthermore, sugar transporter ERDL6 (LOC104891046) participated in the transport and utilization of sugar, was characterized as a drought stress responsive gene in *Arabidopsis* (Kiyosue et al., 1998). In this study, sugar transporter ERDL6 is specifically induced to express in the roots of sugar beet in AS100, which may be involved in resistance to high alkaline salt stress in sugar beet. These results illustrate that these starch and sucrose metabolism DEGs may regulate the mechanical strength of the cell wall to adapt to high alkaline salt environment.

#### DEG_*S*_ Involved in the Sugar Beet Specific Response to High Level Neutral Salt

DNA replication is the biological process by which can exact copy of a DNA molecule is created and genetic information is faithfully transmitted in all living organisms. It is evident that salinity stress affects the cellular molecules involved in DNA replication including helicases, DNA polymerase and DNA replication licensing factor (Sanan-Mishra et al., 2005; John et al., 2016). In our study, transcriptomic analysis also showed that several DEG_*S*_ required for DNA replication process were only up-regulated in the leaves of sugar beet treated with high neutral salt. For example, the expression of seven *minichromosome maintenance (MCM)* (LOC104901820; LOC104904296; LOC109135811; LOC104894762; LOC104901364; LOC104898787; LOC104883739) genes were dramatically increased in NS100. MCM protein functions as DNA replication licensing factor which ensures that DNA in the genome is replicated only once per cell division cycle. Recently, the transcript of pea *MCM6* was reported to be up-regulated in response to salt and cold stress, and over-expression of MCM6 enhanced salinity stress tolerance without affecting yield (Dang et al., 2011). In addition, DNA helicases unwind duplex DNA and participate in the process of DNA replication. A pea *DNA helicase 45* was induced in pea seedlings in response to high salt, and in tobacco plants its over-expression exhibited strong salinity tolerance (Sanan-Mishra et al., 2005). Similarly, *DNA replication ATP-dependent helicase* (LOC104883951), only had specially increasing expression in the leaves of sugar beet in NS100. This report suggested that the DNA replication pathway was specifically involved in sugar beet in response to high neutral salt stress, and provided a new pathway for manipulating neutral salt stress tolerance.

Linoleic acid is known as an important polyunsaturated fatty acid, required for normal plant growth. Lipoxygenase catalyzes the oxygenation of linoleic acid (18:2), which is a key reaction in biosynthesis of plant oxylipins (Vellosillo et al., 2007). It is known that oxylipins have participated in stimulating plant defense gene expression, and play an important role in the adaptation of plants to adverse environments (Jalloul et al., 2002). In this study, the expression of two *linoleate 9S-lipoxygenase 5* (LOC104890834; LOC104890829) was only down regulated in the roots of NS100, which may lead to decreasing the level of oxylipins and tolerance of sugar beet to high neutral salt stress. Furthermore, different from low concentration of neutral salt and alkaline salt, high concentration of neutral salt inhibited the expression of genes related to cutin, suberine and wax biosynthesis, such as *alcohol-forming fatty acyl-CoA reductase* (LOC104901603; LOC104900337) and *omega-hydroxypalmitate O-feruloyl transferase* (LOC104893839). These results indicate that the effect of neutral salt stress on epidermal wax synthesis may lead to growth inhibition in sugar beet.

## Conclusion

In this study, we found a low level of neutral or alkaline salts can enhance photosynthesis and stimulate the growth of sugar beet. Moreover, it indicated that high neutral salt and alkaline salt stresses are different, and the inhibitory effects of high level of neutral salt on sugar beet growth and photosynthesis were greater than those of alkaline salt. On the other hand, the high level of neutral salt induced the hormone content as well as some drastic change in osmotic adjustment substance content. Besides, the pathways of cutin, suberine and wax biosynthesis, sesquiterpenoid, and triterpenoid biosynthesis and flavonoid biosynthesis were involved in low salt treatment enhancing sugar beet growth. Our findings suggest the presence of different mechanisms involved in plant responses to neutral salt and alkaline salt stresses. The pathways of monoterpenoid biosynthesis, amino acids metabolism and starch and sucrose metabolism were specifically enriched in sugar beet at high alkaline salt and related to alkaline salt tolerance of sugar beet. However, high level of neutral salt only significantly affected the expression of genes participating in DNA replication, cutin, suberine and wax biosynthesis, and linoleic acid metabolism compared with high alkaline salt. These findings will enhance our understanding of sugar beet response mechanisms under neutral salt and alkaline salt stresses.

## Data Availability Statement

The datasets analyzed for this study can be found in the National Center for Biotechnology Information (NCBI) and can be accessed in the Short Read Archive (SRA) under the accession number PRJNA634158.

## Author Contributions

GG, YW, RL, ZL, LY, and CL conducted most of the physiological and biochemical analyses. YW, GG, and RL carried out the transcriptome analyses. YW, GG, and PS revised and contributed to writing the manuscript. All authors read and approved the final manuscript.

## Conflict of Interest

The authors declare that the research was conducted in the absence of any commercial or financial relationships that could be construed as a potential conflict of interest.

## Footnotes

1 https://ccb.jhu.edu/software/hisat2/index.shtml

2 http://cufflinks.cbcb.umd.edu/

3 https://github.com/tanghaibao/Goatools

4 http://kobas.cbi.pku.edu.cn/home.do

## Supplementary Material

Supplementary Table 1 The detail of soil used in the experiment.

Supplementary Table 2 List of QRT-PCR primers.

Supplementary Table 3 Sequencing statistics of 30 RNA libraries.

Supplementary Table 4 The FPKM value and annotation of identified genes in the leaves of sugar beet response to neutral salt and alkaline salt treatments.

Supplementary Table 5 The FPKM value and annotation of identified genes in the roots of sugar beet response to neutral salt and alkaline salt treatments.

Supplementary Table 6 The list of differentially expressed genes in the leaves of sugar beet response to neutral salt and alkaline salt treatments.

Supplementary Table 7 The list of differentially expressed genes in the roots of sugar beet response to neutral salt and alkaline salt treatments.

Supplementary Table 8 Gene ontology (GO) enrichment analysis of differentially expressed genes in the leaves of sugar beet response to neutral salt and alkaline salt treatments.

Supplementary Table 9 Gene ontology (GO) enrichment analysis of differentially expressed genes in the roots of sugar beet response to neutral salt and alkaline salt treatments.

Supplementary Table 10 List of selected genes for KEGG pathways in AS25 and NS25.

Supplementary Table 11 List of selected genes for KEGG pathways in AS100.

Supplementary Table 12 List of selected genes for KEGG pathways in NS100.
