# Supplementary material for: Physiological and Transcriptome Analysis of Sugar Beet Reveals Different Mechanisms of Response to Neutral Salt and Alkaline Salt Stresses
Source: Front Plant Sci. 2020 Oct 19;11:571864. doi: 10.3389/fpls.2020.571864 (PMC7604294; doi:10.3389/fpls.2020.571864)
Supplement: Supplementary Table 1 — The detail of soil used in the experiment. [file Table_1.DOCX]

| Sample | Organic matter (%) | inorganic N  (mg kg^-1^) | available P  (mg kg^-1^) | available K  (mg kg^-1^) | Ca  (mg kg^-1^) |
| --- | --- | --- | --- | --- | --- |
| Soil | 3.8±0.11 | 36.9 ±0.21 | 28.9 ±0.14 | 128.1±0.12 | 804.40 ±5.93 |

**Table S1.** The detail of soil used in the experiment
