# Supplementary material for: Physiological and Transcriptome Analysis of Sugar Beet Reveals Different Mechanisms of Response to Neutral Salt and Alkaline Salt Stresses
Source: Front Plant Sci. 2020 Oct 19;11:571864. doi: 10.3389/fpls.2020.571864 (PMC7604294; doi:10.3389/fpls.2020.571864)
Supplement: Supplementary Table 2 — List of QRT-PCR primers. [file Table_2.DOCX]

**Table S2.** List of the QRT-PCR primer

| **Primer name** | **Primer sequence** |
| --- | --- |
| LOC104908747-F | 5-GGTGGAACAACTCGACGTA-3 |
| LOC104908747-R | 5-AGCTCTCTTCAACGTCGTCG-3 |
| LOC104901071-F | 5-GCGCTTACCGACTACTGAGG-3 |
| LOC104901071-R | 5-TGGGCGTTCCACAAATGTCT-3 |
| LOC104904296-F | 5- CGGTGCCCTTGTTTTGAGTG-3 |
| LOC104904296-R | 5- TGCACAAGCCAGTACAGAGG-3 |
| LOC104905367-F | 5-AGGCCATCAAAGAATGGGGG-3 |
| LOC104905367-R | 5-CCGTCACCAAAAAGTGCCTG-3 |
| LOC104892124-F | 5-GTTCTAATGCCGCGAGCTTG-3 |
| LOC104892124-R | 5-AATAACCCACCAGCCCACAC-3 |
| LOC104890829-F | 5-CGCCAGATTGCTCTTAACGC-3 |
| LOC104890829-R | 5-ATCCATGAGGGCTTTTCGGG-3 |
| 18S rRNA-F | 5-CCCCAATGGATCCTCGTTA-3 |
| 18S rRNA-R | 5-TGACGGAGAATTAGGGTTCG-3 |
