# Supplementary material for: Physiological and Transcriptome Analysis of Sugar Beet Reveals Different Mechanisms of Response to Neutral Salt and Alkaline Salt Stresses
Source: Front Plant Sci. 2020 Oct 19;11:571864. doi: 10.3389/fpls.2020.571864 (PMC7604294; doi:10.3389/fpls.2020.571864)
Supplement: Supplementary Table 3 — Sequencing statistics of 30 RNA libraries. [file Table_3.DOC]

**Table S3.** Sequencing statistics of 30 RNA libraries.

| **Sample** | **Total_raw Reads** | **Total_raw Bases** | **Total_clean Reads** | **Total_clean Bases** | **Mapped-reads** | **Mapped-rate (%)** | **Q20%** | **Q30%** | **GC%** |
| --- | --- | --- | --- | --- | --- | --- | --- | --- | --- |
| Control-1 (Leaf) | 55395480 | 8309322000 | 52274056 | 7803557225 | 47318475 | 90.52 | 98.25 | 93.97 | 44.16 |
| Control-2 (Leaf) | 39771810 | 5965771500 | 37227464 | 5550197131 | 33728082 | 90.60 | 98.47 | 94.67 | 45.16 |
| Control-3 (Leaf) | 50593240 | 7588986000 | 46702968 | 6958959142 | 42084044 | 90.11 | 98.1 | 93.61 | 45.07 |
| AS25-1 (Leaf) | 54512642 | 8176896300 | 51357344 | 7661046677 | 47233349 | 91.97 | 98.15 | 93.71 | 44.18 |
| AS25-2 (Leaf) | 50014220 | 7502133000 | 46488098 | 6930549433 | 42192597 | 90.76 | 98.34 | 94.24 | 44.52 |
| AS25-3 (Leaf) | 49845726 | 7476858900 | 46835640 | 6986425896 | 42723470 | 91.22 | 98.32 | 94.18 | 44.57 |
| AS100-1 (Leaf) | 50844772 | 7626715800 | 47242900 | 7044310620 | 42972141 | 90.96 | 98.26 | 94.01 | 44.63 |
| AS100-2 (Leaf) | 49467470 | 7420120500 | 46116608 | 6876480757 | 41919996 | 90.90 | 98.36 | 94.29 | 44.88 |
| AS100-3 (Leaf) | 53715964 | 8057394600 | 50832636 | 7586695803 | 46628777 | 91.73 | 98.28 | 94.05 | 44.29 |
| NS25-1 (Leaf) | 49025018 | 7353752700 | 45125740 | 6733092957 | 41190775 | 91.28 | 98.37 | 94.29 | 45.26 |
| NS25-2 (Leaf) | 52796610 | 7919491500 | 49037114 | 7301481231 | 44790499 | 91.34 | 98.4 | 94.46 | 44.48 |
| NS25-3 (Leaf) | 54309712 | 8146456800 | 50414430 | 7512416041 | 45645224 | 90.54 | 98.24 | 93.99 | 44.74 |
| NS100-1 (Leaf) | 45877810 | 6881671500 | 42742568 | 6371759481 | 39477035 | 92.36 | 98.44 | 94.6 | 44.62 |
| NS100-2 (Leaf) | 50331898 | 7549784700 | 47094792 | 7021476359 | 43623905 | 92.63 | 98.4 | 94.38 | 44.38 |
| NS100-3 (Leaf) | 45479594 | 6821939100 | 42343054 | 6307809177 | 38557584 | 91.06 | 98.54 | 94.87 | 44.54 |
| Control-1 (Root) | 45121060 | 6768159000 | 42567414 | 6357536455 | 38217024 | 89.78 | 98.55 | 94.93 | 43.32 |
| Control-2 (Root) | 43227558 | 6484133700 | 40209808 | 6000126779 | 35368547 | 87.96 | 98.51 | 94.84 | 43.53 |
| Control-3 (Root) | 46778966 | 7016844900 | 42470790 | 6324638889 | 38291664 | 90.16 | 98.44 | 94.62 | 43.76 |
| AS25-1 (Root) | 51008348 | 7651252200 | 46671472 | 6959436313 | 42569049 | 91.21 | 98.65 | 95.22 | 43.6 |
| AS25-2 (Root) | 51321590 | 7698238500 | 47618076 | 7107390088 | 43489588 | 91.33 | 98.59 | 95.07 | 43.53 |
| AS25-3 (Root) | 45263742 | 6789561300 | 41340122 | 6162731871 | 36982873 | 89.46 | 98.49 | 94.77 | 43.63 |
| AS100-1 (Root) | 39047984 | 5857197600 | 36024988 | 5368478860 | 33034913 | 91.70 | 98.5 | 94.78 | 43.59 |
| AS100-2 (Root) | 44766646 | 6714996900 | 40796248 | 6079472417 | 36896126 | 90.44 | 98.49 | 94.78 | 42.93 |
| AS100-3 (Root) | 39089758 | 5863463700 | 35915140 | 5355889244 | 32618130 | 90.82 | 98.5 | 94.8 | 43.12 |
| NS25-1 (Root) | 40727264 | 6109089600 | 37832706 | 5646916663 | 34526127 | 91.26 | 98.53 | 94.89 | 43.35 |
| NS25-2 (Root) | 41049722 | 6157458300 | 35910662 | 5330633098 | 32118496 | 89.44 | 97.5 | 92.25 | 43.62 |
| NS25-3 (Root) | 48331926 | 7249788900 | 43877340 | 6539024954 | 39937154 | 91.02 | 98.44 | 94.66 | 43.67 |
| NS100-1 (Root) | 64079552 | 9611932800 | 60326034 | 9011226655 | 55572342 | 92.12 | 98.53 | 94.86 | 43.27 |
| NS100-2 (Root) | 44060030 | 6609004500 | 40840972 | 6096150514 | 37667628 | 92.23 | 98.44 | 94.65 | 42.9 |
| NS100-3 (Root) | 44361428 | 6654214200 | 39926854 | 5953870644 | 36161751 | 90.57 | 98.61 | 95.11 | 43.2 |
