# Supplementary material for: Physiological and Transcriptome Analysis of Sugar Beet Reveals Different Mechanisms of Response to Neutral Salt and Alkaline Salt Stresses
Source: Front Plant Sci. 2020 Oct 19;11:571864. doi: 10.3389/fpls.2020.571864 (PMC7604294; doi:10.3389/fpls.2020.571864)
Supplement: Supplementary Table 10 — List of selected genes for KEGG pathways in AS25 and NS25. [file Table_10.DOC]

**Table S10.** List of selected genes for KEGG pathways in AS25 and NS25.

|  | **Gene ID** | **Tissues** | **KO** | **A25/CK Log_2_ (fold change)** | **N25/CK Log_2_ (fold change)** |
| --- | --- | --- | --- | --- | --- |
| Cutin, suberine and wax biosynthesis | LOC104901470 | Leaf | Fatty acyl-CoA reductase 3 | +2.6783 | No change |
|  | LOC104900340 | Leaf | Fatty acyl-CoA reductase 3 | No change | +1.36988 |
|  | LOC104900337 | Leaf | Alcohol-forming fatty acyl-CoA reductase | +3.02700 | +2.25482 |
| Sesquiterpenoid and triterpenoid biosynthesis | LOC104908747 | Leaf | beta-amyrin synthase | +2.159298 | +2.292070 |
|  | LOC104901642 | Leaf | Squalene monooxygenase | +1.741566 | +2.490241 |
| Flavonoid biosynthesis | LOC104902217 | Root | Chalcone synthase | -1.097443 | -0.604196 |
|  | LOC104887632 | Root | Chalcone synthase | No change | -1.024976 |
|  | LOC104887630 | Root | Chalcone synthase | No change | -1.393667 |
|  | LOC104905116 | Root | Flavanone 3-dioxygenase | -1.18137298 | No change |
|  | LOC104883093 | Root | Flavonol synthase | -1.044292 | No change |
|  | LOC104886776 | Root | Cytochrome P450 CYP73A100-like | No change | -3.50516 |
