# Supplementary material for: Physiological and Transcriptome Analysis of Sugar Beet Reveals Different Mechanisms of Response to Neutral Salt and Alkaline Salt Stresses
Source: Front Plant Sci. 2020 Oct 19;11:571864. doi: 10.3389/fpls.2020.571864 (PMC7604294; doi:10.3389/fpls.2020.571864)
Supplement: Supplementary Table 11 — List of selected genes for KEGG pathways in AS100. [file Table_11.DOC]

**Table S11.** List of selected genes for KEGG pathways in AS100.

|  | **Gene ID** | **Tissues** | **KO** | **AS100/CK Log_2_ (fold change)** |
| --- | --- | --- | --- | --- |
| Monoterpenoid biosynthesis | LOC104901071 | Leaf | PREDICTED: (3S,6E)-nerolidol/linalool synthase 1 | +2.9196554 |
|  | LOC104888569 | Leaf | PREDICTED: (+)-neomenthol dehydrogenase | +1.77283995 |
| Amino acids metabolism | LOC104890616 | Root | Aspartate-prephenate aminotransferase | -1.282555 |
|  | LOC104892124 | Root | Primary amine oxidase-like | +5.195972944 |
| Starch and sucrose metabolism | LOC104890721 | Root | Pectinesterase 11 | +1.587455207 |
|  | LOC104891046 | Root | Sugar transporter ERDL6 | +1.999942 |
