# Supplementary material for: Physiological and Transcriptome Analysis of Sugar Beet Reveals Different Mechanisms of Response to Neutral Salt and Alkaline Salt Stresses
Source: Front Plant Sci. 2020 Oct 19;11:571864. doi: 10.3389/fpls.2020.571864 (PMC7604294; doi:10.3389/fpls.2020.571864)
Supplement: Supplementary Table 12 — List of selected genes for KEGG pathways in NS100. [file Table_12.DOC]

**Table S12.** List of selected genes for KEGG pathways in NS100.

|  | **Gene ID** | **Tissues** | **KO** | | **NS100/CK Log_2_ (fold change)** |
| --- | --- | --- | --- | --- | --- |
| DNA replication | LOC104901820 | Leaf | | LOW QUALITY PROTEIN: DNA replication licensing factor MCM5 | +1.759197 |
|  | LOC104904296 | Leaf | | DNA replication licensing factor MCM4 | +1.93795538 |
|  | LOC109135374 | Leaf | | probable DNA primase large subunit isoform X1 | +2.170294 |
|  | LOC104903846 | Leaf | | proliferating cell nuclear antigen | +2.229609 |
|  | LOC109135811 | Leaf | | DNA replication licensing factor MCM5-like | +1.70075395 |
|  | LOC104894762 | Leaf | | DNA replication licensing factor MCM7 | +1.216653660 |
|  | LOC104907896 | Leaf | | replication protein A 70 kDa DNA-binding subunit B | +2.301622674 |
|  | LOC104897536 | Leaf | | probable DNA primase large subunit | +1.72729762 |
|  | LOC104883951 | Leaf | | DNA replication ATP-dependent helicase/nuclease DNA2 isoform X1 | +1.9024726951 |
|  | LOC104889194 | Leaf | | DNA polymerase delta small subunit isoform X1 | +1.5667055 |
|  | LOC104886674 | Leaf | | replication protein A 32 kDa subunit A | +1.990978917 |
|  | LOC104901364 | Leaf | | PREDICTED: DNA replication licensing factor MCM2 | +1.965221605 |
|  | LOC104884463 | Leaf | | DNA polymerase alpha catalytic subunit | +1.9692419334 |
|  | LOC104898787 | Leaf | | DNA replication licensing factor MCM6 | +1.9052960501 |
|  | LOC104900347 | Leaf | | DNA polymerase epsilon catalytic subunit A | +1.262423457 |
|  | LOC104883739 | Leaf | | DNA replication licensing factor MCM3 | +2.05316267 |
|  | LOC104892163 | Leaf | | DNA primase small subunit | +1.810747558 |
| Linoleic acid metabolism | LOC104890834 | Root | | probable linoleate 9S-lipoxygenase 5 | -1.0765080 |
|  | LOC104890829 | Root | | probable linoleate 9S-lipoxygenase 5 | -1.09941347 |
| Cutin, suberine and wax biosynthesis | LOC104894616 | Root | | Cytochrome P450 94A1 | -1.3542527 |
|  | LOC104901603 | Root | | Alcohol-forming fatty acyl-CoA reductase | -1.598793485 |
|  | LOC104900337 | Root | | Alcohol-forming fatty acyl-CoA reductase | -1.91091256 |
|  | LOC104893839 | Root | | Omega-hydroxypalmitate O-feruloyl transferase | -1.2398925 |
